# Absorption Amelioration of Amorphous Si Film by Introducing Metal Silicide Nanoparticles

**DOI:** 10.1186/s11671-017-2003-2

**Published:** 2017-03-27

**Authors:** Hui Sun, Hsuan-Chung Wu, Sheng-Chi Chen, Che-Wei Ma Lee, Xin Wang

**Affiliations:** 1 Institute of Materials Science and Engineering, Ocean University of China, Qingdao, 266100 People’s Republic of China; 2 Department of Materials Engineering and Center for Thin Film Technologies and Applications, Ming Chi University of Technology, Taipei, 243 Taiwan; 3 Department of Electronic Engineering, Chang Gung University, Taoyuan, 333 Taiwan

**Keywords:** Absorption ability, Metal silicide, Amorphous silicon, RF sputtering

## Abstract

Amorphous Si (a-Si) films with metal silicide are expected to enhance the absorption ability of pure a-Si films. In this present study, NiSi (20 nm)/Si (40 nm) and AlSi (20 nm)/Si (40 nm) bilayer thin films are deposited through radio frequency (RF) sputtering at room temperature. The influence of the film’s composition and the annealing temperature on the film’s optical absorption is investigated. The results show that all the NiSi/Si films and AlSi/Si films possess higher absorption ability compared to a pure a-Si film (60 nm). After annealing from 400 to 600 °C under vacuum for 1 h, the Si layer remains amorphous in both NiSi/Si films and AlSi/Si films, while the NiSi layer crystallizes into NiSi_2_ phase, whereas Al atoms diffuse through the whole film during the annealing process. Consequently, with increasing the annealing temperature, the optical absorption of NiSi/Si films increases, while that of AlSi/Si films obviously degrades.

## Background

So far, a great deal of research has been done on the development of alternative energies, such as solar energy, since fossil fuels are non-renewable [1–3]. Among the device types of photovoltaic cells, much effort has been put into the thin film solar cells due to their flexible feature, low cost, light weights, etc. [4–6]. As for the film material, amorphous silicon (a-Si) is one of the most popular candidates [7–13]. Their abundance, non-toxicity, low manufacturing cost, uniformity over large areas, and adaptability to various substrates have attracted considerable interest [14, 15]. However, the significant recombination of photo-generate carriers, and the light-induced degradation derived from the Staebler-Wronski effect limit the widespread use of a-Si in photovoltaic applications [16–18]. Decreasing the film’s thickness is an effective solution to solve these problems [19, 20]. But unfortunately, the thinner film will possess a low optical absorption and the photoelectric conversion efficiency is also suppressed.

In order to enhance the optical absorption of a-Si thin film, many approaches have been explored, for instance, the introduction of metallic nanoparticles [21–23], dielectric nanopillars [24], and addition antireflection coatings [25, 26]. Sachan et al. demonstrated a strategy of embedding metal silicide nanoparticles into an ultrathin a-Si film [27]. The optical absorption was found to be greatly improved in the visible range (350–750 nm). Brahmi et al. also reported that silicide films exhibit high absorption coefficient in the range of 5 × 10^5^–10 × 10^5^ cm^−1^ in the visible light region, even though their thickness is ultrathin (~10 nm) [28].

In this work, we have introduced Ni and Al into an a-Si thin film so as to embed Ni silicide or Al silicide in the Si film. The influence of metal (Ni or Al) content on the film’s optical behavior is investigated. The effect of annealing temperature on the film’s microstructure and optical properties is also discussed.

## Methods 

a-Si thin films with Ni silicide or Al silicide were deposited by radio frequency sputtering on glass and silicon substrates at room temperature. Firstly, NiSi layers or AlSi layers were co-sputtered from the Ni target or Al target and Si target. The layer’s thickness was maintained at 20 nm. The target power on Si was varied in order to adjust the film’s composition (shown in Table 1). Then, a top Si layer (40 nm) was deposited over the NiSi and AlSi layers. A pure Si film with a total thickness of 60 nm was also prepared for the purpose of comparison. Finally, all the films were annealed under vacuum at 400, 500, and 600 °C for 1 h, respectively.

**Table 1:** Nomenclature for Ni silicide or Al silicide nanoparticle-embedded Si thin films

Composition	NS-1	NS-2	NS-3	AS-1	AS-2	AS-3
Si (at.%)	45	56	71	42	52	69
Ni (at.%)	55	44	29	–	–	–
Al (at.%)	–	–	–	58	48	31

The film’s composition was measured by JEOL JXA-8200 electron probe X-ray microanalyzer (EPMA). The phase formation was identified by Raman spectroscopy (iHR 550) with a 532-nm laser. The depth-profiling analysis was obtained by Auger electron spectroscopy (AES, ULVAC-PHI, PHI 700). The microstructures of the specimens were observed on the film’s cross-section by high-resolution transmission electron microscopy (HR-TEM, JEOL JEM-2100). The film’s absorption coefficient was estimated by the following equation (Eq. 1) from the film’s transmittance and reflectance data, which were measured by UV-VIS spectrophotometer (JASCO-V670).

1 $$ \alpha =\frac{1}{d} \ln \left(\frac{1- R}{T}\right) $$

where *d* is the film thickness, *R* and *T* are the optical reflectance and transmittance, respectively.

## Results and Discussion

**Fig. 1:**
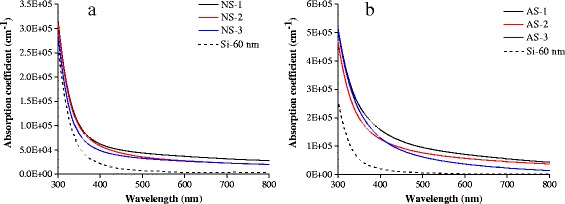
The absorption coefficient of **a** NiSi/Si and **b** AlSi/Si films with various compositions

**Fig. 2:**
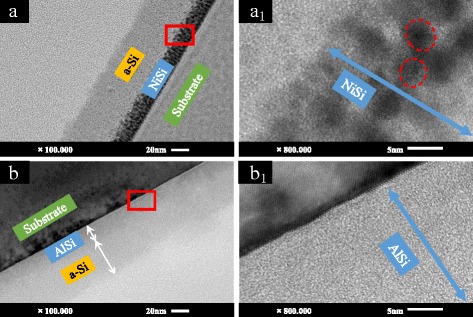
The cross-sectional TEM images of **a** NiSi/Si (NS-1) and **b** AlSi/Si (AS-1) films and high-resolution images of **a**_**1**_ NiSi/Si (NS-1) and **b**_**1**_ AlSi/Si (AS-1) films derived from the areas marked with *red rectangles* in Fig. 2a, b

The absorption coefficient of NiSi/Si films and AlSi/Si films is shown in Fig. 1. With increasing Ni content or Al content in the films, their absorption ability is significantly enhanced in the whole visible region (400–800 nm). Especially, compared with the pure Si film, the films with Ni silicide or Al silicide possess a much higher absorption coefficient (10^4^~10^5^ cm^−1^) at the wavelength above 500 nm, where the absorption of the visible light by the pure Si film is negligible. The absorption enhancement of NiSi/Si and AlSi/Si films is believed to be attributable to the plasmonic absorption by Ni silicide or Al silicide nanoparticles in an a-Si matrix [27]. These results confirm that an obvious improvement in absorption can be achieved within amorphous Si films. Additionally, with respect to the NiSi/Si film, this improvement for the AlSi/Si film is much more evident. It may be caused by the coarse-grained interface between the NiSi layer and Si layer introducing more scattering of visible light (shown in Fig. 2). In contrast, the smooth interface between the AlSi layer and Si layer is beneficial for improving the film’s absorption ability. Since the band gap of amorphous silicon is about 1.7 eV, a significant light absorption of the a-Si film can be found below 730 nm, which lies within the visible light region. Thus, the absorption enhancement of NiSi/Si and AlSi/Si films can significantly improve the efficiency of PV devices based on amorphous silicon.

Figure 2 shows the cross-sectional images of NiSi/Si (NS-1) and AlSi/Si (AS-1) films. The NiSi layer or AlSi layer and the top Si layer can be clearly observed. In the high-resolution image of Fig. 2a_1_, some nanoparticles assumed to be Ni silicide are noted. They lead the microstructure of the first layer to be much coarser. However, in the AlSi/Si film, Al silicide nanoparticles are imperceptible.

**Fig. 3:**
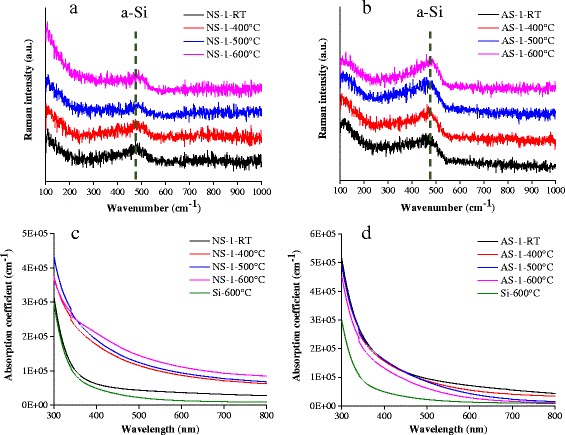
Raman spectra of **a** NiSi/Si film and **b** AlSi/Si film before and after annealing in vacuum at 400, 500, and 600 °C, and the absorption coefficient of **c** NiSi/Si films and **d** AlSi/Si films annealed at different temperatures

The Raman spectra of the NiSi/Si film and AlSi/Si film before and after annealing under vacuum at 400, 500, and 600 °C is shown in Fig. 3a, b, respectively. Whatever the annealing temperature, the NiSi/Si film and AlSi/Si film display a pure amorphous Si structure peaked at 480 cm^−1^. No peak near 520 cm^−1^ assigned to the crystalline Si structure is detected. The absorption coefficient of NiSi/Si and AlSi/Si films annealed at different temperatures is shown in Fig. 3c, d, respectively. The absorption ability of the NiSi/Si film greatly enhances with increasing the annealing temperature, while that of AlSi/Si films gradually decreases. Since the dopants in Si are generally activated through an essential thermal annealing treatment between 400 and 1000 °C [29, 30], the degradation of the absorption ability of AlSi/Si films proves that the NiSi/Si film is more suitable for the fabrication of the absorbing layer in amorphous silicon solar cells.

**Fig. 4:**
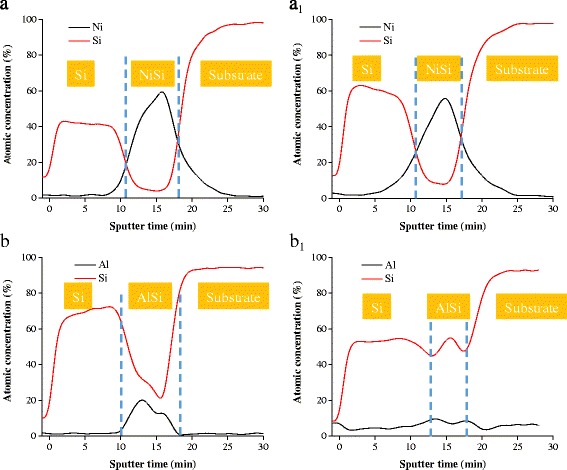
The depth profile of atomic composition analysis of **a**, **b** as-deposited NiSi/Si (NS-1) and AlSi/Si (AS-1) films and **a**_**1**_, **b**_**1**_ those films annealed at 500 °C

The depth profiles of the atomic composition analysis of as-deposited NiSi/Si and AlSi/Si films and those films annealed at 500 °C are given in Fig. 4. After 1 h annealing under vacuum, the composition of the Si layer and NiSi layer remains nearly unchanged (Fig. 4a, a_1_). Few nickel atoms diffuse from the NiSi layer into the adjacent Si layer. In contrast, as for the AlSi/Si film, almost all the aluminum atoms in the AlSi layer are diffused. The Al content in the AlSi layer and that in the top Si layer are close. This perhaps explains why the absorption ability of the AlSi layer is suppressed after annealing. The uniformly distributed Al clusters in the whole film reinforce the scattering and reflection of the visible light.

**Fig. 5:**
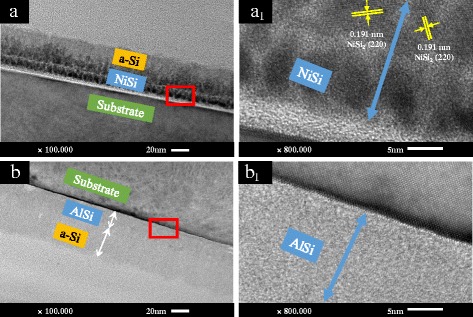
The cross-sectional TEM images of **a** NiSi/Si (NS-1) and **b** AlSi/Si (AS-1) films annealed at 500 °C and high-resolution images of **a**_**1**_ NiSi/Si (NS-1) and **b**_**1**_ AlSi/Si (AS-1) films derived from the areas marked with *red rectangles* in Fig. 5a, b

The cross-sectional images of NiSi/Si (NS-1) and AlSi/Si (AS-1) films annealed at 500 °C are shown in Fig. 5. Some nickel silicide nanoparticles are crystallized to form NiSi_2_ compounds after annealing. NiSi_2_ (220) planes with interplanar lattice spacing of 0.191 nm are identified (Fig. 5a_1_). The strengthening of absorption ability of NiSi/Si films with annealing temperature increasing can be due to the amelioration of the film’s crystallinity. Besides, the interface between the AlSi layer and top a-Si layer becomes illegible after annealing (Fig. 5b). No crystallized phase can be detected (Fig. 5b_1_). This is attributed to the diffusion of aluminum atoms during the annealing process.

## Conclusions

In this work, nickel silicide and aluminum silicide nanoparticles are introduced into amorphous Si thin films in order to enhance the film’s absorption ability. Radio frequency sputtering was used to deposit NiSi/Si and AlSi/Si bilayer thin films. The results show that all the NiSi/Si and AlSi/Si films present an absorption improvement, especially in the long wavelength region (>500 nm), compared to the pure amorphous Si film. The as-deposited AlSi/Si films possess higher absorption compared with the NiSi/Si films. However, the AlSi/Si film’s absorption ability significantly degrades after 1 h annealing under vacuum condition, which is owing to the diffusion of aluminum atoms during the annealing process, whereas the optical absorption of NiSi/Si films gradually improves after annealing. This is resulting from the enhanced crystallinity of NiSi/Si films. Our results confirm that the amorphous Si film with suitable metal silicides can expand the response to the visible light of the photovoltaic devices and improve the utilization of the solar spectrum.

## Abbreviations

AES: Auger electron spectroscopy
EPMA: Electron probe X-ray microanalyzer
HR-TEM: High-resolution transmission electron microscopy
RF: Radio frequency
